# Intraoperative Electron Beam Radiotherapy (IOERT) in the management of locally advanced or recurrent cervical cancer

**DOI:** 10.1186/1748-717X-8-80

**Published:** 2013-04-08

**Authors:** Brandon M Barney, Ivy A Petersen, Sean C Dowdy, Jamie N Bakkum-Gamez, Kristi A Klein, Michael G Haddock

**Affiliations:** 1 Department of Radiation Oncology, Mayo Clinic, 200 First Street SW, Rochester, MN, 55905, USA; 2 Division of Gynecologic Surgery, Mayo Clinic, Rochester, MN, USA

**Keywords:** Intraoperative radiotherapy, Cervix cancer, Locally advanced, Recurrent, Pelvic relapse

## Abstract

**Background:**

To report outcomes in women with locally recurrent or advanced cervical cancer who received intraoperative electron beam radiotherapy (IOERT) as a component of therapy.

**Methods:**

From 1983 to 2010, 86 patients with locally recurrent (n = 73, 85%) or primary advanced (n = 13, 15%) cervical cancer received IOERT following surgery. Common surgeries included pelvic exenteration (n = 26; 30%) or sidewall resection (n = 22; 26%). The median IOERT dose was 15 Gy (range, 6.25-25 Gy). Sixty-one patients (71%) received perioperative external beam radiotherapy (EBRT; median dose, 45 Gy). Forty-one patients (48%) received perioperative chemotherapy.

**Results:**

Median follow-up was 2.7 years (range, 0.1-25.5 years). Resections were classified as R0 (n = 35, 41%), R1 (n = 30, 35%), or R2 (n = 21, 24%). Cumulative incidences of central (within the IOERT field) and locoregional relapse at 3 years were 23 and 38%, respectively. The 3-year cumulative incidence of distant relapse was 43%. Median survival was 15 months, and 3-year Kaplan-Meier estimates of cause-specific (CSS) and overall survival (OS) were 31 and 25%, respectively. On multivariate analysis, pelvic exenteration (p = 0.02) and perioperative EBRT (p = 0.009) were associated with improved central control in patients with recurrent disease. Recurrence within 6 months of initial therapy was associated with reduced CSS (p = 0.001). Common IOERT-related toxicities included peripheral neuropathy (n = 16), ureteral stenosis (n = 4), and bowel fistula/perforation (n = 4). Eleven of 16 patients with neuropathy required long-term pain medication.

**Conclusions:**

Long-term survival is possible with combined modality therapy including IOERT for advanced cervical cancer. Distant relapse is common, yet a significant number of patients experienced local progression in spite of aggressive treatment. In addition to consideration of disease- and treatment-related morbidity, other factors to be considered when selecting patients for this approach include the time interval from initial therapy to recurrence and whether the patient is able to receive perioperative EBRT and pelvic exenteration in addition to IOERT.

## Background

The incidence of cervical cancer in the United States continues to decline, with an estimated 12,170 cases of invasive cancer to be diagnosed in 2012 [1]. Unfortunately, cervical cancer continues to be the leading cause of cancer mortality in women in developing countries, resulting in approximately 190,000 deaths per year [2]. While the prognosis for early-stage disease is excellent, with 5-year survival rates approaching 90% [3], the prognosis for locally advanced disease with pelvic sidewall or locoregional lymph node involvement is poor [4,5]. This is also true for women with locally recurrent cancer, where poor prognostic factors include pelvic sidewall fixation, early recurrence after primary therapy, and tumor recurrence >3 cm in greatest dimension [6-8].

The importance of achieving local control in patients with cervical cancer cannot be overemphasized, as more than half of recurrences after primary therapy are limited to the pelvis [9]. Furthermore, persistent pelvic disease may result in significant morbidity including pain, anorexia, vaginal bleeding, cachexia, and/or psychological problems [10], and approximately 60% of women who die of cervical cancer have local failure as the major cause of death [11]. Retrospective studies of radiation alone have shown a dose–response relationship for pelvic disease control in locally advanced cervical cancer; [12] however, the use of higher radiation doses to improve tumor control may result in significant morbidity, particularly when reirradiation is attempted in the salvage setting [13]. Intraoperative radiotherapy (IORT) has been used at our institution and at others to give a focally-targeted radiotherapy boost at the time of surgery to areas of close or positive surgical margins to maximize radiotherapy dose while minimizing irradiation of normal tissues [14-24]. Here we update a previous report with long-term outcomes from the largest single-institution series of women with locally advanced or recurrent cervical cancer treated with a combined modality approach that includes IORT.

## Methods

The Mayo Clinic Institutional Review Board (IRB) approved this study. We queried the prospectively-maintained IORT database for patients with cervical carcinoma treated with IORT at Mayo Clinic, Rochester, MN from 1980 to 2010. All patients were treated with a combined modality approach that included, at a minimum, surgery and IORT. Patients selected for this approach had either locally advanced primary cervix cancer with pelvic wall extension and/or extensive paraaortic (PA) lymph node (LN) involvement or locally recurrent cervix cancer within the pelvis and/or abdomen. In addition to having pathologically-confirmed disease, patients underwent a multidisciplinary evaluation that included a radiation oncologist and a gynecologic oncologist. In cases where chemotherapy was deemed appropriate, a medical oncologist was also involved in all decision making.

Details regarding the administration of IORT at Mayo Clinic have been described previously [25] and are summarized as follows. IORT is delivered in a dedicated operating suite. All patients in this study were treated using high-energy electrons (IOERT) from a linear accelerator. IOERT was delivered employing one of a series of custom-made Lucite collimating devices of various lengths, shapes, and diameters, selected to best encompass the at-risk field. The dose was prescribed to the 90% isodose level such that ≥90% of the prescription dose was delivered to the margin in question. Resection margins were graded based on the both the surgeon’s subjective assessment and the frozen pathologic assessment prior to the delivery of IOERT as R0 (microscopically negative), R1 (microscopically positive), or R2 (grossly positive). Dose was selected based on the amount of residual disease and proximity of critical structures. Routine follow-up after IOERT consisted of pelvic examination every three months with surveillance imaging of the abdomen and pelvis every six months for two years. Follow-up thereafter consisted of physical examination with imaging annually or as indicated by symptoms.

Clinical data including patterns of failure, survival, and toxicity were recorded prospectively in the IORT database through patient visits or contact with local physicians. All endpoints were defined from the date of IOERT. Determination of disease progression was made based on radiographic and/or physical exam findings. Central control (CC) was defined as freedom from recurrence within the IOERT field. Locoregional control (LRC) was defined freedom from recurrence within the IOERT field as well as locoregional lymph nodes. Freedom from distant relapse (FFDR) was defined as freedom from relapse in sites outside the pelvis and paraaortic region. Toxicity was scored using the NCI Common Toxicity Criteria (CTCAE) v.4.

The Kaplan-Meier (KM) method was used to determine cause specific (CSS) and overall survival (OS). The cumulative incidence methodology was used to calculate rates of central relapse (CR), locoregional relapse (LRR) and distant relapse (DR). For patients with recurrent cervical cancer, the Log-Rank test was used to assess an array of variables for potential impact on clinical outcomes. These variables included tumor grade (low vs. high), history of previous radiotherapy (RT; yes vs. no), multiple previous recurrences (yes vs. no), time period during which IOERT occurred (1983–1996 vs. 1997–2010), time from initial diagnosis to recurrence treated with IOERT (≤6 months vs. >6 months), tumor size prior to IOERT (<3 cm vs. ≥3 cm), type of surgery performed in conjunction with IOERT (pelvic exenteration vs. less invasive surgery), surgical margin grade prior to delivery of IOERT (R0 vs. R1 vs. R2), pelvic sidewall involvement by tumor (yes vs. no), perioperative external beam radiotherapy +/− brachytherapy (EBRT +/− BT; yes vs. no), and perioperative chemotherapy (yes vs. no). Variables associated with outcomes by the Log-Rank test were then assessed using a multivariate Cox proportional hazard model controlled for patient age. These variables included tumor grade, time from initial diagnosis to recurrence treated with IOERT, history of previous RT, type of surgery performed in conjunction with IOERT, surgical margin grade, perioperative RT, and perioperative systemic therapy. In all cases, a *P* value of <0.05 was considered significant. All statistical analysis was performed with JMP 8.0 (SAS Institute Inc., Cary, NC, USA).

## Results

### Patient and treatment characteristics

Of the 86 patients who met criteria for inclusion in this study, 73 (85%) had locally recurrent cervical cancer and 13 (15%) had locally advanced primary disease. The median age at IOERT was 48.6 years (range, 20.9-85.5 years). Most patients had squamous cell carcinoma histology (n = 68, 79%), and the majority of tumors were high-grade (n = 73, 85%). The median tumor size was 5.0 cm (range, 0.5-14.0 cm). Table 1 summarizes patient and tumor characteristics at the time of IOERT.

**Table 1:** Patient and tumor characteristics at the time of recurrence treated with IOERT

**Characteristic**	**Value (%)**
Patients (n)	86
Age (y)	
Median	48.6
Range	20.9-85.5
Locally advanced primary disease (n)	13/86 (15)
Recurrent disease (n)	73/86 (85)
First recurrence	59/73 (81)
Multiple previous recurrences	14/73 (19)
Previous EBRT +/− BT	59/73 (81)
Previous surgery	58/73 (79)
Previous systemic therapy	30/73 (41)
Interval from diagnosis to recurrence (mo)	
Median	25.0
Range	3.1-379.4
Tumor size in maximum dimension (cm)	
Median	5.0
Range	0.5-14.0
Tumor histology (n)	
SCC	68 (79)
AC	11 (13)
ASC	6 (7)
CC	1 (1)
Tumor grade (n)	
High	73 (85)
Low	8 (9)
Unknown	5 (6)

Abbreviations: EBRT = external beam radiotherapy; BT = brachytherapy; SCC = squamous cell carcinoma; AC = adenocarcinoma; ASC = adenosquamous carcinoma; CC = clear cell carcinoma.

In the 73 patients with recurrent disease, the median time from initial cancer diagnosis to recurrent treated with IOERT was 2.1 years (range, 0.3-31.6 years). Fourteen (19%) of those patients had previously experienced at least one previous local recurrence prior to IOERT. Therapy at the time of initial diagnosis in patients with recurrent disease consisted of primary surgery +/− chemotherapy (n = 23, 31%), primary EBRT and BT +/− chemotherapy (n = 18, 25%), or surgery and EBRT +/− chemotherapy (n = 32, 44%). In all, 59 patients (81%) with recurrent disease had previously received RT, 58 (79%) had previously undergone some form of surgery, and 30 (41%) had previously received systemic therapy. In the 13 patients with locally advanced primary disease, primary therapy consisted of surgery and EBRT in addition to IOERT, with 10 of 13 patients (77%) receiving adjuvant chemotherapy.

Treatment characteristics are summarized in Table 2. The most common surgeries performed in conjunction with IOERT included pelvic exenteration (n = 26, 30%) and pelvic sidewall resection (n = 22, 26%), each occurring with or without lymph node dissection (LND). Other surgeries included paraaortic LND only (n = 17, 20%), exploration only (n = 12, 14%), radical hysterectomy/LND (n = 7, 8%), abdominal wall resection (n = 1, 1%), and inguinal LND (n = 1, 1%). In all, 58 patients (67%) were found to have tumor involving the pelvic sidewall. The most common surgical margin grade after maximal debulking was R0 (n = 35, 41%), but patients commonly had either microscopic (R1; n = 30, 35%) or gross (R2; n = 21, 24%) residual disease at the time of IOERT. The median IOERT dose was 15 Gy (range, 6.25-25 Gy), and the most commonly treated field was the hemipelvis (n = 53, 61%). The most common electron beam energy was 9 MeV (n = 44, 51%), and most patients (n = 74, 86%) were treated with a single IOERT field.

**Table 2:** Treatment characteristics

**Characteristic**	**Value (%)**
Surgery	
Type of resection (n)	
Exenteration	26 (30)
Pelvic sidewall resection	22 (26)
Paraaortic LND	17 (20)
Exploration	12 (14)
Radical hysterectomy/LND	7 (8)
Abdominal wall resection	1 (1)
Inguinal LND	1 (1)
Resection status (n)	
R0	35 (41)
R1	30 (35)
R2	21 (24)
IOERT	
IOERT dose (Gy)	
Median	15
Range	6.25-25
IOERT field (n)	
Hemipelvis	53 (61)
Paraaortic	22 (26)
Bilateral pelvis	6 (7)
Aortic bifurcation	4 (5)
Groin	1 (1)
IOERT energy (MeV)	
6	3 (4)
9	44 (51)
12	26 (30)
15	5 (6)
18	8 (9)
IOERT cone size (cm)	
Median	7
Range	5-15
Total IOERT fields (n)	
1	74 (86)
2	11 (13)
3	1 (1)
Perioperative EBRT +/− BT	
Timing (n)	
Pre-operative	47 (55)
Post-operative	12 (14)
Pre- and post-operative	2 (2)
None	25 (29)
Dose (Gy)	
Median	45
Range	19.8-83
Perioperative chemotherapy (n)	
Concurrent	15 (17)
Sequential	26 (31)
None	45 (52)

Abbreviations: LND = lymph node dissection; IOERT = intraoperative electron beam radiotherapy; EBRT = external beam radiotherapy; BT = brachytherapy.

In addition to surgical debulking and IOERT, 61 patients (71%) received peri-operative EBRT +/− BT, including 35 patients with recurrent disease who had been previously irradiated. EBRT was most commonly administered preoperatively, though some patients were treated postoperatively or both pre- and postoperatively depending on surgical findings. BT techniques changed over the time interval in question from low-dose (LDR) to high-dose rate (HDR) implants. The median dose for all patients treated with perioperative RT was 45 Gy (range, 19.8-83 Gy). For patients with recurrent disease who had previously received RT, the median retreatment dose was 39.6 Gy (range, 20–54 Gy), although generally, any obviously overlapping fields were kept to a cumulative dose of ≤80 Gy. Some patients (n = 41, 48%) received perioperative chemotherapy in addition to surgery and IOERT. This was given concurrently with EBRT in 15 patients and sequentially in 26 patients. Systemic regimens varied over the study time interval but universally consisted of cisplatin-containing combination therapy.

### Treatment outcomes

**Figure 1:**
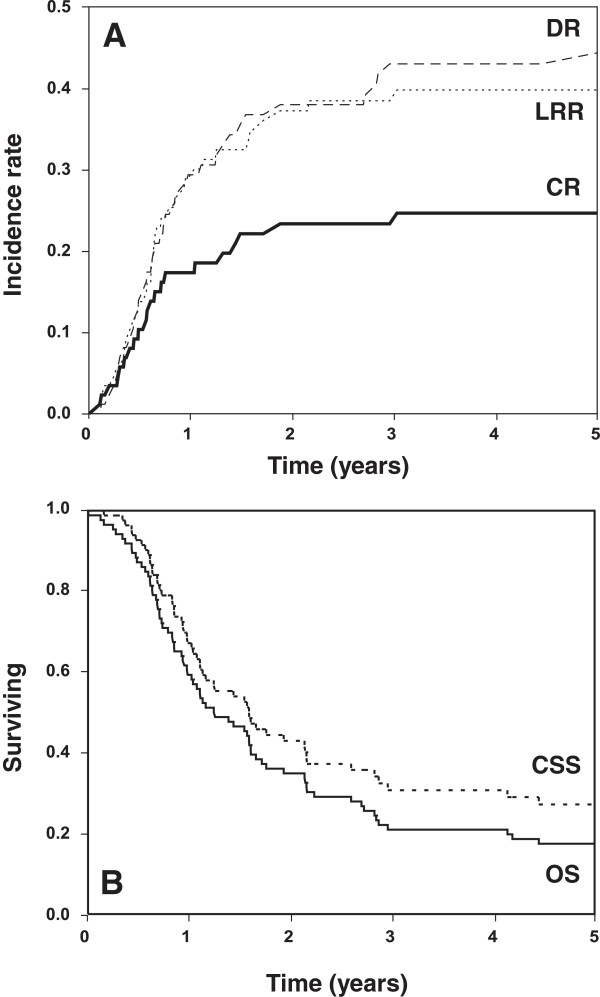
Cumulative incidence of central relapse (CR), locoregional relapse (LRR), and distant relapse (DR; A) and cause-specific (CSS) and overall survival (OS; B).

Median follow-up in living patients was 2.7 years (mean, 5.2 years; range, 0.1-25.5 years), and the median survival was 15 months. Cumulative incidences of CR, LRR and DR were 23, 38 and 43%, respectively, at 3 years (Figure 1A). Likewise, 3-year estimates of CSS and OS were 31 and 25% (Figure 1B). For patients with locally advanced primary disease, 3-year cumulative incidences of CR, LRR and DR were 22, 30 and 27%. For patients with recurrent cervical cancer, 3-year cumulative incidences of CR, LRR and DR were 23, 39 and 44%.

On univariate analysis, no factor was found to be prognostic for CR or LRR in patients with recurrent cervical cancer (Table 3). Surgical margins were associated with DR (p = 0.004), with 3-year cumulative DR rates of 33, 44 and 51% for patients who underwent R0, R1, and R2 resections, respectively. This association was also true for CSS (p = 0.04), where 3-year estimates of CSS for R0, R1, and R2 resections were 45, 27, and 14%. The time interval from initial diagnosis to recurrence treated with IOERT was strongly associated with CSS (p = 0.001), and no patient recurring within 6 months of initial diagnosis survived 3 years. Risk factors for which no association could be established by Log-Rank testing included tumor grade, history of previous RT, history of multiple recurrences, time period during which IOERT occurred, size of tumor recurrence, type of surgery, tumor location, delivery of perioperative RT, and treatment with perioperative chemotherapy.

**Table 3:** Univariate analysis of potential prognostic risk factors in patients treated with IOERT for recurrent cervical cancer

**Variable**	**3-year CC, %**	***P***	**3-year LRC, %**	***P***	**3-year FFDR, %**	***P***	**3-year CSS, %**	***P***
Tumor grade		0.68		0.61		0.46		0.21
Low	92		50		71		59	
High	65		47		42		25	
Previous RT		0.22		0.61		*0.08*		0.28
Yes	71		54		50		33	
No	66		46		37		26	
Multiple previous recurrences		0.95		0.20		0.77		0.72
Yes	65		45		50		32	
No	71		54		47		31	
Time period of IOERT		0.16		0.13		0.63		0.45
1983-1996	60		40		45		27	
1997-2010	81		68		50		39	
Time from diagnosis to recurrence		0.76		0.47		0.13		**0.001**
≤6 months	80		60		25		0	
>6 months	70		52		49		34	
Tumor size		0.67		0.97		0.46		0.82
<3 cm	44		35		42		22	
≥3 cm	74		55		48		34	
Type of surgery		0.13		0.12		0.60		0.30
Exenteration	77		58		46		41	
Less extensive resection	66		49		48		27	
Surgical margin grade		0.17		0.13		**0.004**		**0.04**
R0	82		71		61		45	
R1	55		36		45		27	
R2	81		53		25		14	
Pelvic sidewall involvement		0.77		0.76		0.89		0.37
Yes	69		50		46		28	
No	70		55		48		39	
Perioperative RT		0.13		0.11		0.34		0.29
Yes	76		60		44		36	
No	52		33		60		22	
Perioperative systemic therapy		0.35		0.97		0.44		0.81
Yes	74		54		53		31	
No	66		51		43		32	

Abbreviations: CC = central control; LRR = locoregional control; FFDR = freedom from distant recurrence; CSS = cause-specific survival; RT = radiotherapy; IOERT = intraoperative electron beam radiotherapy.

Multivariate analysis revealed pelvic exenteration (p = 0.02) and perioperative RT (p = 0.009) to be associated with improved CC (Table 4). The same was true for LRC, where both pelvic exenteration (p = 0.02) and perioperative RT (p = 0.01) predicted for improved LRC. Surgical margin grade was associated with FFDR, as both R0 (p = 0.004) and R1 (p = 0.02) resections predicted for improved FFDR compared to R2 resections; however, there was no association when R0 and R1 resections were directly compared. Factors associated with poor CSS included high tumor grade (p = 0.04) and recurrence within 6 months of initial diagnosis (p = 0.002). No other factor was statistically association with CSS, though trends for improved CSS were seen in patients who had an R0 (p = 0.07) or R1 (p = 0.08) resection compared to an R2 resection or who received perioperative RT (p = 0.07).

**Table 4:** Multivariate analysis of potential prognostic risk factors in patients treated with IOERT for recurrent cervical cancer

**Variable**	**CC**			**LRC**			**CSS**		
	**HR**	**95% CI**	***P***	**HR**	**95% CI**	***P***	**HR**	**95% CI**	***P***
High grade tumor	0.92	0.17-3.97	0.91	0.78	0.24-2.14	0.64	0.40	0.14-0.96	**0.04**
Recurrence ≤6 months after initial therapy	0.24	0.03-4.88	0.28	0.32	0.08-2.15	0.21	0.10	0.03-0.40	**0.002**
Previous RT	2.21	0.57-9.00	0.25	1.26	0.43-3.51	0.66	1.40	0.60-3.20	0.43
Pelvic exenteration	4.18	1.15-20.62	**0.02**	3.01	1.19-8.55	**0.02**	1.75	0.83-3.90	0.15
Surgical margin grade									
R0 vs. R2	0.69	0.13-3.52	0.65	1.60	0.56-4.61	0.38	2.25	0.94-5.42	*0.07*
R1 vs. R2	0.54	0.12-1.90	0.35	1.20	0.47-2.89	0.69	2.02	0.92-4.34	*0.08*
R0 vs. R1	1.29	0.34-5.61	0.72	1.33	0.51-3.64	0.56	1.11	0.50-2.50	0.79
Perioperative RT	5.54	1.52-23.86	**0.009**	3.38	1.30-9.19	**0.01**	2.09	0.93-4.67	*0.07*
Perioperative systemic therapy	0.98	0.33-3.10	0.97	0.74	0.33-1.63	0.45	0.86	0.44-1.69	0.66

### Toxicity

Toxicity graded as being potentially related to delivery of IOERT (effects that may not have occurred if IOERT had been excluded from treatment) was reported by 30 patients (35%). The most serious was a bowel perforation that led to a patient’s death. Other serious side effects included one grade 4 bowel perforation as well as cases of ureteral stenosis (n = 4), abscess formation (n = 2), fistula formation (n = 2), peripheral neuropathy (n = 1), hemorrhage (n = 1), and severe soft tissue fibrosis (n = 1), each grade 3. In all, 16 patients (19%) experienced some degree of peripheral neuropathy, and 11 of these (13%) required some form of long-term prescription pain medication.

## Discussion

In this study, we report the outcomes of patients with cervical carcinoma treated with IOERT. We found that long-term survival is possible with combined modality therapy including IOERT for advanced cervical cancer, even in women with poor prognostic factors, such as pelvic sidewall disease or large tumor size. While distant relapse after salvage therapy was a common pattern in this population, a significant number of patients still experienced local recurrence in spite of aggressive treatment. This finding underscores the challenging nature of treating advanced cervical cancer.

Treatment for recurrent cervical cancer is usually a function of initial cancer therapy, site of recurrence, and patient performance status and comorbidity. In patients treated with primary RT who experience a vaginal apex or paravaginal tissue recurrence without pelvic sidewall involvement, salvage rates with radical hysterectomy approach 40% in appropriately-selected patients [26,27]. Patients treated with primary surgery who experience a vaginal cuff recurrence have a similar salvage rate when treated with concurrent RT and cisplatin-based chemotherapy [6,28]. Pelvic exenteration is typically reserved for more extensive pelvic recurrences involving the bladder, lower vagina, and/or rectum and can result in long-term survival in a small percentage of patients [29-32]. When tumor involves the pelvic sidewall, as is the case in the majority of patients in this study, salvage outcomes are universally poor, and resection of these recurrences is generally considered to be futile [6,7]. In the 58 patients with pelvic sidewall involvement treated with IOERT in this study, 3-year rates of CC, LRC, and CSS were 69, 50, and 25%, respectively, indicating that a small but quantifiable number of patients with pelvic sidewall involvement can be salvaged with a combined modality approach that includes IOERT.

Previously reported factors prognostic for improved clinical outcomes in patients with recurrent cervical cancer include: disease-free interval >6 months, recurrent tumor <3 cm in size, and no pelvic sidewall fixation [10]. On univariate analysis, we found that disease-free interval >6 months was associated with improved CSS (p = 0.001), but neither tumor size nor pelvic sidewall involvement were predictive of clinical outcomes. Interestingly, surgical margin grade (R0 vs. R1 vs. R2) was prognostic for FFDR (p = 0.004) and CSS (p = 0.04) but did not appear to influence CC or LRC. This finding was confirmed on the multivariate analysis, where both R0 (p = 0.004) and R1 (p = 0.02) resections were associated with improved FFDR, but no correlation was found between surgical margin status and local disease control. The finding that surgical margin status is predictive of distant rather than local progression has been previously reported by our group [15] and could be due to a number of factors. Firstly, an R2 resection may be indicative of more extensive local disease which could in turn predict for a higher likelihood of occult distant spread, and these patients may succumb to distant metastasis before local recurrence is detected. It is also possible that cutting through tumor during an R2 resection may result in dissemination of tumor cells throughout the abdominal cavity, promoting distant recurrence. Indeed, some groups have reported a rare but quantifiable incidence of cervical cancer port site recurrences after laparoscopic surgery, implying peritoneal spread is possible with this malignancy [33]. Finally, cervical squamous cell carcinoma (SCC) may be more radiosensitive than other pelvic malignancies [34], which could result in patients with SCC benefitting more from IORT than those with other tumors. Thus, the impact of surgical margin status on disease control within the IORT field would be minimal, but IORT would have no effect on systemic disease control. This last scenario makes the strongest argument for including IORT as a component of treatment for locally advanced or recurrent SCC of the cervix.

Table 5 summarizes the published literature on IORT for cervical cancer [14,16-24]. Clinical outcomes from the current series, including crude rate of locoregional relapse, median survival, and OS compare favorably with those reported from other groups. Our study represents the largest of its kind, both for total number of patients and number of patients with recurrent cervical cancer treated with IORT. Three other large series of note include two French studies, one of which is a single-institution experience (n = 54) [17] and the other a multi-institutional effort (n = 70) [19], and one other from Spain (n = 66) [20]. Outcomes in patients with recurrent disease in the single-institutional French study and the Spanish study were similar to those in the current study, but the French multi-institutional group reported significantly worse survival (8% at 3 years) [19]. Potential reasons for this may be that only 43% of patients in that study received EBRT in addition to IORT, only 43% of patients underwent an R0/R1 resection, and/or patients were treated at one of several institutions where the IORT procedure may not have been common practice. Aside from the current study, the most recent series comes from Stanford University [22], and includes 17 patients with cervical cancer among 36 total patients with recurrent gynecologic malignancies. They report excellent outcomes in women with recurrent cervical cancer at 5 years, with 45% LRC and 46% CSS. One potential reason for these seemingly superior outcomes at 5 years is that only 32% of the patients from the Stanford series had pelvic sidewall involvement, compared to 67% in the current series. Additionally, 18% of the patients in the Stanford series underwent exenteration, while 30% had an exenteration in our study, implying a more advanced disease state in the patients treated at our institution. Another interesting difference between these studies is that the Stanford data showed no benefit to exenteration compared to less extensive surgery, while in our series, exenteration predicted for improved CC and LRC. The reason for this difference is unclear but may in part be due to sampling error, as only 7 patients underwent exenteration in the Stanford series. Overall, the median survival after IORT in the studies summarized in Table 5 ranged from 7–27 months, and OS ranged from 8-54% at 3 years.

**Table 5:** Literature review of IORT for cervical cancer

**Series**	**Date**	**Patients (n)**	**Advanced primary or recurrent**	**IORT modality**	**Median IORT dose (Gy)**	**Crude LRR**	**Median survival (mos)**	**OS**
Hicks et al [18].	1993	13	Recurrent	Orthovoltage	15	7/13	7	-
Konski et al [24].	1993	8	Primary	IOERT	20	7/8	27	-
		5	Recurrent	IOERT	20	-	9	-
Gerard et al [17].	1994	20	Primary	IOERT	-	4/20	-	75% at 18 mos
		34	Recurrent	IOERT	-	6/34	-	32% at 4 yrs
Stelzer et al [23].	1995	22	Recurrent	IOERT	22	10/22	26	43% at 5 yrs (CSS)
Mahé et al [19].	1996	70	Recurrent	IOERT	19	50/67	11	8% at 3 yrs
del Carmen et al [14].	2000	5	Recurrent	IOERT	15	2/5	-	-
Gemignani et al [16].	2001	9	Recurrent	HDR-BT	14	-	-	54% at 3 yrs
Martinez-Monge et al [20].	2001	31	Primary	IOERT	12	6/31	-	58% at 10 yrs
		36	Recurrent	IOERT	15	18/36	-	14% at 10 yrs
Roth et al [21].	2003	1	Recurrent	HDR-BT	15	0/1	20	-
Tran et al [22].	2007	17	Recurrent	Orthovoltage	11.5	-	-	47% at 5 yrs (CSS)
**Current series**	**2012**	**13**	**Primary**	**IOERT**	**12.5**	**4/13**	**13**	**29% at 3 yrs**
		**73**	**Recurrent**	**IOERT**	**17.5**	**33/73**	**17**	**25% at 3 yrs**

Abbreviations: IORT = intraoperative radiotherapy; LRR = locoregional recurrence; OS = overall survival; IOERT = intraoperative electron beam radiotherapy; CSS = cause-specific survival; HDR-BT = high-dose rate brachytherapy.

## Conclusions

While the current study is retrospective in nature and subject to the usual elements of bias and uncertainty, it provides evidence to support the concept that a small but significant proportion of women with locally advanced or recurrent cervical cancer may experience long-term survival after combined modality therapy including IOERT. Because treatment-related morbidity with this approach can be severe, it must be balanced against both the likelihood of cure and the risk of morbidity from untreated local disease. Good candidates for combined modality therapy with IOERT include women with a disease-free interval >6 months who will tolerate pelvic exenteration if necessary. If possible, perioperative RT with concurrent chemotherapy should be strongly considered, even in previously irradiated patients, as the delivery of perioperative RT was associated with improved LRC. Patients who do not meet these criteria should be considered for a less aggressive approach that may involve palliative RT and/or surgery, along with palliative chemotherapy.

## Competing interests

The authors declare that they have no competing interests.

## Authors’ contributions

Dr. BMB queried the institutional database, performed statistical analysis, and wrote the manuscript. Drs. MGH and IAP are the radiation oncology faculty who treated the great majority of the patients with intraoperative radiotherapy and were also involved in writing the manuscript. Drs. SCD and JNB-G are the gynecologic oncologists who dictated management strategies and operated on a number of these patients and were also involved in manuscript authorship. KAK was involved in data collection and maintenance. All authors read and approved the final manuscript.
